# Development of a Cyclodextrin-Based Drug Delivery System to Improve the Physicochemical Properties of Ceftobiprole as a Model Antibiotic

**DOI:** 10.3390/ijms26135953

**Published:** 2025-06-20

**Authors:** Dariusz Boczar, Wojciech Bocian, Jerzy Sitkowski, Karolina Pioruńska, Katarzyna Michalska

**Affiliations:** 1 Department of Synthetic Drugs, National Medicines Institute, Chełmska 30/34, 00-725 Warsaw, Poland; 2 Laboratory for Analysis of Bioactive Compounds, Institute of Organic Chemistry, Polish Academy of Sciences, Kasprzaka 44/52, 01-224 Warsaw, Poland; wo.bocian@gmail.com; 3 Falsified Medicines and Medical Devices Department, National Medicines Institute, Chełmska 30/34, 00-725 Warsaw, Poland; j.sitkowski@nil.gov.pl (J.S.); k.piorunska@nil.gov.pl (K.P.)

**Keywords:** ceftobiprole, cyclodextrin, drug delivery, lyophilisation, SBE-β-CD, ternary system

## Abstract

This study presents a methodology for developing a cyclodextrin-based delivery system for ceftobiprole, a poorly water-soluble and amphoteric drug, chemically stable in acidic conditions. Ceftobiprole is a broad-spectrum cephalosporin antibiotic administered clinically as its water-soluble prodrug, ceftobiprole medocaril, due to limited aqueous solubility of the parent compound. Solubility enhancement was achieved through complexation with anionic sulfobutylether-β-cyclodextrin (SBE-β-CD). At a pH below 3, ceftobiprole is protonated and cationic, which facilitates electrostatic interactions with the anionic cyclodextrin. An optimised high-performance liquid chromatography (HPLC) method was used to assess solubility, the impurity profile, and long-term chemical stability. X-ray powder diffraction (XRPD) confirmed the amorphous nature of the system and the absence of recrystallization. Nuclear magnetic resonance (NMR) and attenuated total reflection Fourier transform infrared (ATR-FTIR) spectroscopy supported the formation of a host–guest complex. The freeze-dried system prepared from 0.1 M formic acid solution contained negligible residual acid due to nearly complete sublimation. The most promising formulation was a ternary system of ceftobiprole, maleic acid, and SBE-β-CD (1:25:4 molar ratio), showing ~300-fold solubility improvement, low levels of degradation products, and stability after eight months at −20 °C. After pH adjustment to a parenterally acceptable level, the formulation demonstrated solubility and a pH comparable to the marketed drug product.

## 1. Introduction

Among the various approaches aimed at improving the bioavailability of drugs with low solubility or permeability, included in the Biopharmaceutical Classification System (BCS) classes II and IV, complexes with cyclodextrins (CDs) have attracted great attention [1,2,3,4,5,6,7,8]. In general, CDs comprise a macrocyclic ring composed of glucose residues linked by α-1,4-glycosidic bonds. This arrangement results in a remarkable three-dimensional (3D) structure resembling a truncated cone, with hydroxylic groups located on the outer surface. Consequently, the cavity of a CD becomes hydrophobic, allowing the poorly water-soluble drugs to enter, while the presence of many OH groups on the outer surface should allow a drug–CD complex to improve its solubility [1]. In relation to antibacterial drugs and antibiotics, our research group demonstrated the advantages of preparing CD inclusion complexes including slowing down the degradation of an active pharmaceutical ingredient (API), modifying the drug release profile, improving the permeability through biological membranes, and increasing the antimicrobial activity [9]. It was observed that the cavity size of β-CD, composed of seven glucose residues, is appropriate to form the inclusion complexes with the majority of APIs [10]. As each glucose residue contains three OH groups, they may react with different substituents, often in an uncontrolled manner, thus resulting in a mixture of CD derivatives differing in the degree of substitution (DS), i.e., the number showing how many hydroxyl groups underwent the reaction. For example, sulfobutyl ether (SBE) of β-CD has been approved by the European Medicines Agency (EMA) as a drug excipient suitable for oral and parenteral administration [11].

**Scheme 1:**
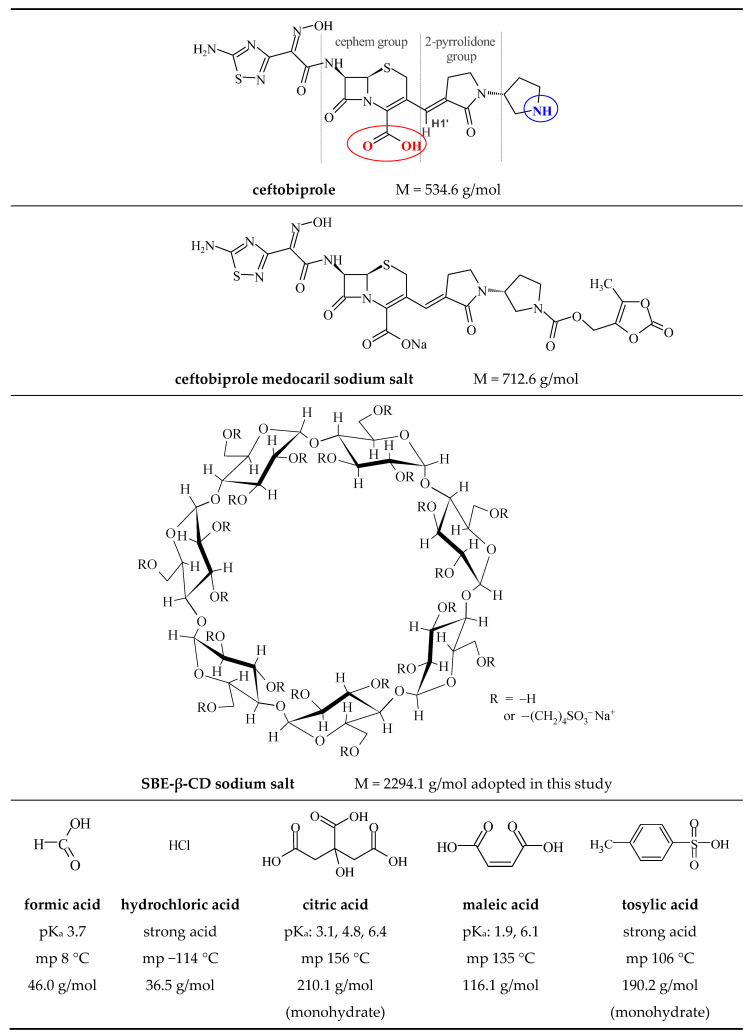
Structural formulas of the compounds discussed in this paper. For ceftobiprole, the predicted ionizable function groups and the H1′ atom were marked. For acids, the pK_a_ values, melting points (mp), and molar masses are given.

By the end of 2021, there were 34 reports of CD complexes with cephalosporins (a subgroup of β-lactam antibiotics) with a significant improvement in solubility demonstrated for cefuroxime axetil, cefixime, cefpodoxime proxetil, and cefdinir [9]. Among cephalosporins, ceftobiprole is a relatively novel antibiotic, approved and authorised in some countries in 2018 in the form of a prodrug with a covalently attached medocaril group (Scheme 1). The use of native ceftobiprole is limited due to its low solubility. The medicinal product Zevtera introduced to the market takes advantage of fact that the medocaril prodrug is characterised by significantly greater solubility compared to active ceftobiprole, which allows the drug to be administered to the patient at a concentration of 2 mg/mL. It is assumed that the prodrug undergoes biotransformation in vivo to the proper drug; however, it should be emphasised that the efficiency of metabolic reactions may vary from person to person and may decrease with age [12]. Therefore, in this study, a new drug delivery system was developed using the active form of the drug, ceftobiprole, instead of its prodrug, ceftobiprole medocaril.

According to theoretical calculations, ceftobiprole is an amphoteric compound that exists predominantly in a neutral form at pH values above 3, with a dissociated carboxyl group and a protonated secondary amino group [13]. At pH values below the pK_a_ of the carboxyl group of ceftobiprole (3.0 or 3.2, depending on the software used), the –COOH group remains largely undissociated, resulting in a cationic, protonated form of ceftobiprole. Molecular docking and molecular dynamics (MD) simulations were performed for 18 different combinations of CDs (β-CD, HP-β-CD and SBE-β-CD), ceftobiprole protonation states (non-ionised, protonated and zwitterionic) and orientations (two opposite arrangements inside the CD cavity). These simulations indicated that the thermodynamically most stable complexes, characterised by the highest negative value of free Gibbs energy, were formed with anionic SBE-β-CD under acidic conditions, where ceftobiprole is protonated [13]. Because protonated ceftobiprole favourably interacts with anionic SBE-β-CD, thereby increasing solubility, it was necessary to acidify the solution to a pH well below 3. Our previous studies [14] demonstrated that ceftobiprole degrades relatively slowly in acidic environments compared to other tested stress conditions. The $t_{90}$ value, representing the time required for a 10% decrease in the initial concentration, was approximately 22 h in 0.1 M HCl at room temperature. In contrast, degradation was significantly faster under UV irradiation at 366 nm ($t_{90}$ ≈ 1 h), in 3% H_2_O_2_ ($t_{90}\;$ ≈ 25 min), and in 0.01 M NaOH ($t_{90}$ ≈ 10 min) [14]. Moreover, another kinetic study revealed that the rate constants for both acidic degradation in 0.1 M HCl at 25 °C and thermal degradation in water at 45 °C decreased 2.5-fold in the presence of 10 mM SBE-β-CD [13].

The aim of this study was to develop new freeze-dried cyclodextrin-based drug delivery systems for ceftobiprole—a model substance that is amphoteric, relatively stable in acidic environment, and poorly soluble in water. The systems were developed with SBE-β-CD, which was selected as the best carrier based on MD simulations. The goal was to obtain physically and chemically stable systems with satisfactory physicochemical properties in relation to the currently registered formulation of the drug. The properties of the new systems were characterised using a range of analytical techniques, including an optimised high-performance liquid chromatographic (HPLC) method [14], nuclear magnetic resonance (NMR), X-ray powder diffraction (XRPD) and attenuated total reflectance infrared Fourier transform (ATR-FTIR) spectroscopy. Long-term chemical stability and impurity profiles were assessed by HPLC or liquid chromatography coupled to tandem mass spectrometry (LC-MS/MS), while XRPD analysis was used to confirm the physical stability of the obtained systems. This goal may nevertheless be more universal, as the proposed methodology can be used to develop a drug delivery system with increased solubility in water for a basic or an amphoteric compound, which is thermally unstable but chemically stable in acidic solutions.

## 2. Results and Discussion

### 2.1. Selection of Volatile and Non-Volatile Acids for Ionisation of Ceftobiprole

The newly developed systems were prepared by freeze-drying the solutions containing ceftobiprole and SBE-β-CD in a solvent characterised by an acidic pH. The acid of choice had to be a small and hydrophilic molecule; hence, it could therefore not show a strong affinity for the CD cavity and thus could not compete with ceftobiprole. In order to find the most appropriate one, several acids differing in strength and volatility (with structural formulas and selected physicochemical properties depicted in Scheme 1) were tested. The results of the performed experiments are summarised in Table 1.

When an acid is a liquid at room temperature, it should sublimate from the frozen solution at a sufficiently low pressure, similar to water, completely or partially leaving the sample. Therefore, performing the freeze-drying of solutions containing liquid acids should have the advantage that the pH of the solution is decreased when the complexation equilibrium is reached (allowing the most favourable conditions for complex formation) but the solid freeze-dried product should be deprived of the acidic additive to a high extent. The first solvent containing a volatile acid used for freeze-drying was a 0.1 M aqueous solution of formic acid, which is the simplest liquid organic acid, starting a homologous series of monocarboxylic acids. Herein, the molar ratio of ceftobiprole and SBE-β-CD was set to 1:2. Although the computational studies of this complex were performed assuming 1:1 stoichiometry [13], the excess of SBE-β-CD was applied to achieve a shift of the dynamic equilibrium towards complex formation. The next volatile acid that was used in this study was hydrochloric acid, which is 100% dissociated. HCl is a small molecule that does not compete with ceftobiprole for inclusion in the CD cavity. The mixture of ceftobiprole and SBE-β-CD was freeze-dried in 0.01 M HCl, because its pH ≈ 2 should be sufficient for the protonation of most ceftobiprole molecules.

Another approach is to use a solid acid, which allows the formation of a ternary system. The acid does not sublimate but remains in the system, causing the presence of the API in the form of a salt. Out of the anions encountered in FDA-approved drug products [15,16], citrate, tosylate and maleate were selected as three anions of solid acids. Citric acid (triprotic with a lowest pK_a_ of 3.1), a representative of weak acids, appeared a promising additive since it is widely used as an acidity regulator, and constitutes an excipient in the Zevtera drug product [17]. According to the Henderson–Hasselbach equation, the pH of the solution should be much lower than the pK_a_ for the carboxyl group of ceftobiprole to ensure that a considerable proportion of API molecules is present in its protonated, cationic form. When preparing the solution intended for freeze-drying, a 20-fold molar excess of citric acid in relation to ceftobiprole caused a decrease in pH only to 2.9; a 50-fold excess lowered the pH to 2.7, while a 100-fold molar excess was needed to obtain a pH of 2.5. Ultimately, it was decided to prepare the system in a molar ratio of 1:100:1 (ceftobiprole:citric acid:SBE-β-CD).

Maleic acid is a diprotic acid with pK_a_ values of 1.9 and 6.1. The relatively low value of the first pK_a_ (1.9) indicates that it is a medium-strength acid, undergoing acidic dissociation to a large extent but not 100%. A series of experiments with varying molar ratio of ceftobiprole to maleic acid was performed to establish its impact on the solubility of ceftobiprole, starting from 1:5 and ending at 1:25 (Table 1). Another solid acid used in this study was *p*-toluenesulfonic acid, further referred to as tosylic acid, which is strong, monoprotic and solid. The initial solution containing ceftobiprole, tosylic acid and SBE-β-CD in proportions of 1:25:2 was characterised by pH 2.4, sufficient to obtain a desirable extent of ceftobiprole protonation.

### 2.2. Solubility and Chemical Stability Studies Using HPLC

The selective HPLC method [14] optimised by our research group allowed us to determine the solubility of ceftobiprole in all prepared systems (Table 1).

**Figure 1:**
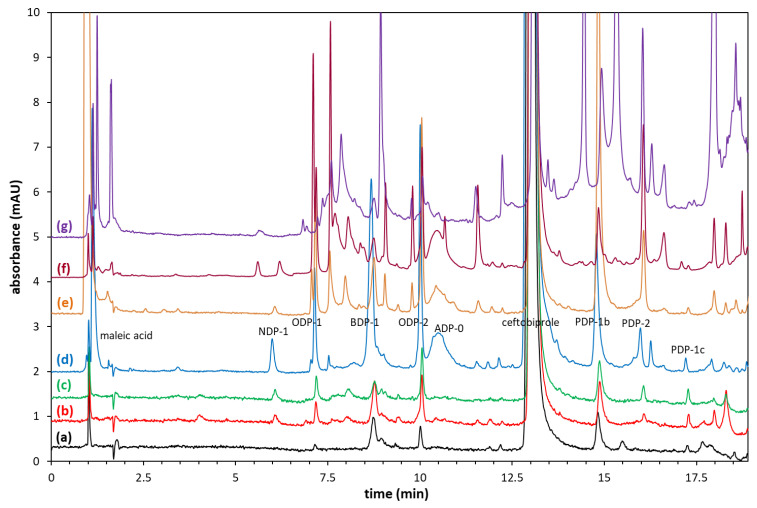
Chromatograms recorded at 320 nm in the preliminary studies for aqueous solutions of: (**a**) standard of ceftobiprole; and drug delivery systems prepared by freeze-drying, arranged with increasing detected DPs; (**b**) ceftobiprole in 0.1 M HCOOH; (**c**) ceftobiprole/SBE-β-CD 1:2 in 0.1 M HCOOH; (**d**) ceftobiprole/maleic acid/SBE-β-CD 1:25:4 in H_2_O; (**e**) ceftobiprole/citric acid/SBE-β-CD 1:100:1 in H_2_O; (**f**) ceftobiprole/tosylic acid/SBE-β-CD 1:25:2 in H_2_O; (**g**) ceftobiprole/SBE-β-CD 1:2 in 0.01 M HCl. Peak heights were normalised to allow more readable visualisation.

**Scheme 2:**
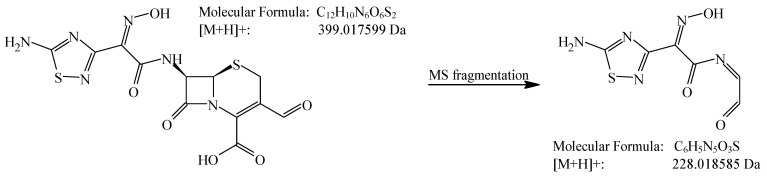
The proposed molecular structure of NDP-1 and its main fragment ion detected in LC-MS/MS.

Chromatograms of the selected delivery systems of ceftobiprole are shown in Figure 1. The identity of individual degradation products (DPs) was confirmed by comparing the retention times with the chromatograms of samples subjected to forced degradation under stress conditions [14], as well as by performing the LC-MS/MS analysis. For the freeze-dried samples, a novel DP (NDP-1) was detected, which was not observed in the previous degradation studies [14]. Scheme 2 presents the proposed molecular structure of NDP-1 together with the proposed structure for the main fragment ion detected in the fragment mass spectrum. NDP-1 forms most probably as a result of the oxidation of a double bond linking the cephem and 2-pyrrolidone groups with the formation of an aldehyde. This mechanism of degradation is different from the degradation pathways described in [14]. The LC-MS data also provided information on the composition of the SBE-β-CD used in this study, which is an important result because this randomly substituted CD may differ from batch to batch. The interpretation of the deconvoluted mass spectrum (Figure S1, Table S1) is provided in Supplementary Materials.

The samples were stored in a freezer, taking into account the recommended storage conditions for ceftobiprole. The stability of the prepared samples was tested at defined intervals using the same methodology. To evaluate potential changes in solubility due to physical transformations such as phase transitions or recrystallization, an identical volume of water was added as in the initial solubility test. Subsequently, the reconstituted samples were analysed by HPLC to determine the precise solubility and to monitor any increase in the concentration of DPs content, thereby assessing both physical and chemical stability.

#### 2.2.1. Systems with Volatile Acids

The solubility of unprocessed ceftobiprole in 0.1 M HCOOH was approximately 0.14 mg/mL [13]. In contrast, ceftobiprole subjected to freeze-drying in the same solvent achieved a 15-fold higher solubility (2.1 mg/mL, Table 1). As discussed earlier, when the solution pH is much lower than the pK_a_ of the carboxyl group, ceftobiprole should be mostly protonated, which means acquiring an overall charge of +1. It seems that these cations repel each other, thus preventing the formation of multi-molecular agglomerates, which in turn would preferably form at a higher pH, when ceftobiprole would have an overall charge of zero. In an acidic environment, when the aqueous solvent disappears from the sample due to sublimation, the individual ceftobiprole molecules remain at a certain distance from each other, which results in a more developed product surface, larger volume and lower density. When the powder freeze-dried in an acidic environment is then dissolved in water, the solvent-accessible surface is considerably increased, resulting in significantly higher solubility. Therefore, it seems to us that the use of freeze-drying in an acidic environment allowed us to overcome the intermolecular interactions between ceftobiprole molecules, thus inhibiting their aggregation.

**Figure 2:**
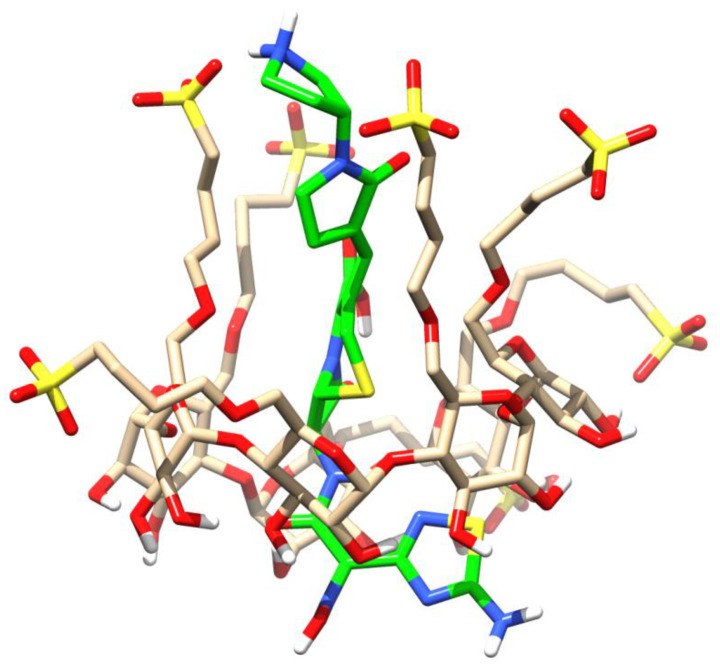
The most probable structure of the complex between protonated ceftobiprole and SBE-β-CD, taken from [13].

The freeze-drying of ceftobiprole with the addition of SBE-β-CD (in a molar ratio of 1:2) further increased the solubility of ceftobiprole to 3.4 mg/mL. The increase in solubility caused by the addition of SBE-β-CD can be explained based on the shape of the inclusion complex obtained in MD studies (Figure 2) [13]. Sulfobutyl ether chains align parallel to the ceftobiprole molecule due to favourable electrostatic attraction between the SO_3_^−^ groups of SBE-β-CD and the positively charged secondary amine group of ceftobiprole, thus protecting it from the undesirable contact with its surrounding [13].

A freeze-drying experiment was also performed in 0.01 M HCl. The resulting powder was intensely yellow compared to the slightly yellow powder from 0.1 M HCOOH, suggesting that ceftobiprole degradation took place during or after the ~40-h lyophilisation. Although HPLC indicated a concentration of ceftobiprole of 1 mg/mL, gravimetric estimation suggested ~10 mg/mL. This discrepancy, along with numerous DP peaks in the chromatogram (Figure 1g), confirms significant degradation. The reconstituted solution showed a pH of ~1.0 (vs. ~2.0 initially), suggesting that some acid remained in a solid sample, unlike in the HCOOH sample, where the pH rose from 2.3 to 4.0. After months of storage in the freezer, the 0.01 M HCl-derived sample became a brown, viscous solid. These findings indicate incomplete HCl removal and ongoing solid-state degradation; therefore, HCl was excluded from further studies.

#### 2.2.2. Systems with Non-Volatile Acids

A preliminary study of the ceftobiprole/citric acid/SBE-β-CD system (1:100:1) showed a solubility of 8.6 mg/mL. The plasticity of the resulting solid, in contrast to the coarse-grained 0.1 M HCOOH sample, likely reflects its hygroscopic nature. Despite consistent dissolution of the same sample mass in water, the ceftobiprole concentration decreased to 7.3 mg/mL after 6 months and 5.1 mg/mL after 9 months of storage, likely due to moisture uptake. Consequently, citric acid was deemed unsuitable for ceftobiprole formulation, and further development was discontinued.

In contrast to the weak acidic strength of citric acid, tosylic acid was selected, which is a model of a strong acid occurring in a solid state at room temperature. The dominant disadvantage of this system is too rapid a degradation in the solid state. The consistency of the powder remained coarse-grained, but over time the powder became increasingly yellow. In all stability tests, the concentration determined by the HPLC decreased rapidly with the time—the initial 21.3 mg/mL dropped to 4.5 mg/mL after 9 months (for further details, see Table 1). The impurity profile revealed the presence of known as well as unknown DPs, the latter being characteristic for this system (Figure 1f). In addition, significant amounts of ADP-5 and ADP-6 were detected, indicating advanced progress of acidic degradation, according to the results presented in [14]. Due to the lack of long-term stability of the system with tosylic acid, no further studies were continued using it.

The most promising results were obtained with maleic acid. To assess its impact on ceftobiprole solubility, experiments were conducted at a fixed ceftobiprole:SBE-β-CD molar ratio of 1:4, with varying maleic acid excess. As a reference, several analogues without SBE-β-CD were prepared. As shown in Table 1, higher maleic acid content correlated with lower pre-lyophilisation pH, and increased ceftobiprole solubility after reconstitution. Notably, despite similar initial pH values (~2.5 for 25-fold molar excess of maleic acid vs. ~2.3 for 0.1 M HCOOH), maleic acid enabled significantly higher solubility (14 mg/mL vs. 3.4 mg/mL). This indicates that initial pH alone does not determine solubility; the final acidic environment after reconstitution is equally critical. The samples containing solid acids became even more acidic (approximately 1.5 after dissolution) compared to the pH of the starting solutions before freeze-drying (Table 1) due to the stacking of acid concentration. After the first portion of water was added to the freeze-dried sample, the solid acid dissolved instantly, thus creating a strongly acidic environment. This allowed ceftobiprole to dissolve even more easily, making it possible to achieve concentrations exceeding 10 mg/mL. Among the series of experiments performed with maleic acid and SBE-β-CD, the greatest solubility of ceftobiprole was obtained for the molar ratio of 1:25:4 (about 14 mg/mL in a preliminary test). Further increase of the maleic acid content would result in excessive acidification of the samples.

Importantly, additional experiments showed that both the use of (i) freeze-drying, (ii) decreasing the pH well below 3, and (iii) SBE-β-CD are necessary to obtain such high solubility. The effect of freeze-drying was tested by examining the solubility of a physical mixture for the same components: ceftobiprole, maleic acid and SBE-β-CD, in the same molar ratio of 1:25:4, obtaining 1.3 mg/mL for the physical mixture mimicking the complex, and only 0.7 mg/mL for the 1:25 mixture of ceftobiprole and maleic acid. These results clearly demonstrated that the use of the freeze-drying technique allowed for a 10-fold better solubility of ceftobiprole compared to simply mixing the components. The role of the acidic environment is even more profound. Freeze-drying of pure aqueous solutions of ceftobiprole or its mixture with SBE-β-CD in a molar ratio of 1:4 resulted in relatively poor solubilities—0.12 and 0.15 mg/mL, respectively. This clearly shows that an appropriately low pH is necessary to increase solubility. We hypothesise that, similarly to 0.1 M HCOOH, its effect is most likely manifested by reducing the degree of agglomeration of ceftobiprole molecules. In turn, pure water allows the formation of ceftobiprole aggregates, thereby eliminating the benefits of freeze-drying. The third important component of the process allowing for such a large improvement in solubility is the presence of SBE-β-CD, which enables a several-fold increase in solubility compared to the analogous system without CD (Table 1).

Solid-state stability studies were conducted for 8 months. The solubility of the tested samples did not change, meaning that the same weight was successfully dissolved in the same volume of water. Over the 8 months, the sum of DPs in the discussed 1:25:4 system with maleic acid increased by only 0.5%, but a decrease in the ceftobiprole concentration was observed from 14.0 to 11.3 mg/mL. The reason for this situation may be (i) the underestimation of the DPs content, or (ii) the hygroscopicity of the tested sample, which is manifested by the absorption of water from the air by the sample in order to return to its original hydrated state. As a result, the sample mass should increase and therefore the percentage of ceftobiprole should decrease, which is observed as a decrease in the ceftobiprole concentration over time. For laboratory purposes, small-scale, multiple cycles involving heating the sample (freezer-room temperature), opening, closing and freezing the hygroscopic substance may contribute to uncontrolled water absorption, and in this way we can observe an increase in the content of impurities in the sample.

### 2.3. NMR Studies on Complex Formation

The significant improvement of ceftobiprole solubility achieved by freeze-drying in an acidic environment (without this, the water solubility of ceftobiprole is 0.05 mg/mL) allowed for NMR studies of samples dissolved in water. Measurements were performed only for the two selected systems, i.e., one system with a volatile acid (HCOOH) and one with a solid acid (maleic acid).

**Figure 3:**
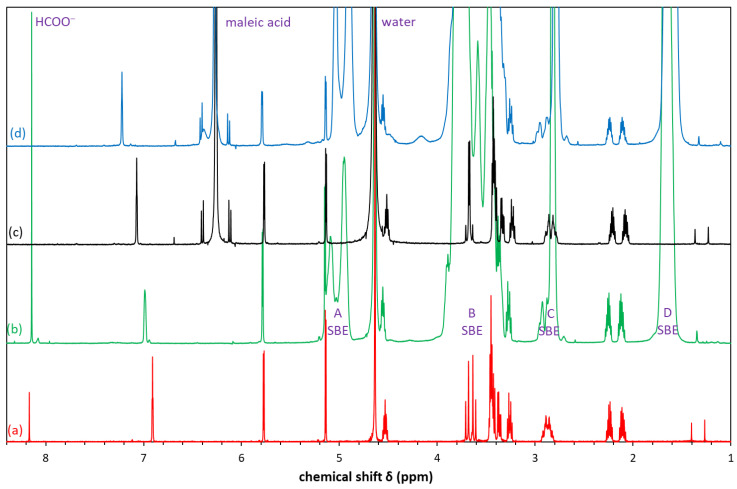
^1^H NMR spectra in D_2_O of the following freeze-dried samples: (**a**) ceftobiprole in 0.1 M HCOOH; (**b**) ceftobiprole/SBE-β-CD 1:2 complex in 0.1 M HCOOH; (**c**) ceftobiprole/maleic acid 1:25 in H_2_O; (**d**) ceftobiprole/maleic acid/SBE-β-CD 1:25:4 in H_2_O. The concentration of ceftobiprole was set to 2 mg/mL (3.75 mM) for (**a**) and 4 mg/mL (7.5 mM) for (**b**–**d**). The interpretation of multiplets A–D from SBE-β-CD was discussed in [18].

In the recorded ^1^H NMR spectra (Figure 3), the signals characterised by the highest chemical shifts δ are of greatest importance. The singlet at 8.1 ppm is attributed to the formate anions remaining in the samples which were freeze-dried in 0.1 M HCOOH. In addition to the characteristic chemical shift, this peak is also characterised by a ^1^J CH coupling constant of 210 Hz, which can be determined from the distance between its carbon satellites. Since the intensity of each peak in the ^1^H NMR spectrum is proportional to the concentration of corresponding equivalent protons, this property allows us to determine the molar ratio of formates to ceftobiprole, given that the area of the well-separated signal of ceftobiprole at about 7 ppm is set to 1. When ceftobiprole was freeze-dried in 0.1 M HCOOH, the proportion of formate anions in the sample was determined to be about 0.27 (Figure 3a), while for the corresponding complex with SBE-β-CD it rose to about 0.77 (Figure 3b). These results confirmed that the formic acid, initially used in 600-fold molar excess with respect to ceftobiprole in order to form an acidic environment before freezing the solution, sublimated almost completely during freeze-drying, but a certain non-stoichiometric amount remained in the sample and hence was detected in the ^1^H NMR spectra.

The observed presence of residual formic acid was further confirmed by the pH measurements of the dissolved samples. In the case of the two discussed samples freeze-dried in HCOOH, the pH was approximately 5 (without SBE-β-CD) and 4 (with SBE-β-CD), which is in good agreement with the NMR results—the more residual formic acid, the lower the pH of the solution. The rather unexpected result that the amount of the residual HCOOH was non-stoichiometric is probably the result of two opposing processes occurring during the freeze-drying process. On the one hand, the electrostatic attraction between the protonated, positively charged ceftobiprole and the formate anions would tend to leave some formate anions in the sample due to electrostatic interactions. On the other hand, formic acid tends to sublimate during freeze-drying and thus leave the sample in the same manner as water. From this reasoning, it follows that the presence of SBE-β-CD resulted in a higher degree of retention of formate anions in the freeze-dried sample.

The signal at about 7 ppm can be unambiguously assigned to a H1′ proton from a CH group which links the cephem group and 2-pyrrolidone ring (Scheme 1), belonging to the system of four conjugated double bonds. For this signal, two differences were observed for samples freeze-dried in HCOOH with and without SBE-β-CD: the chemical shift changed from 6.91 to 6.99 ppm and the signal was broadened. The chemical shift value is due to the chemical surrounding of the analysed proton [19]; therefore, the observed variability suggests an interaction with another species located in close proximity to this proton, leading to a change in the charge distribution in the conjugated bond system or, less likely, a significant change in the local geometry of the molecule when the sample contains SBE-β-CD. This may confirm the formation of a complex between ceftobiprole and CD. Other ^1^H NMR signals of ceftobiprole did not change their chemical shifts significantly in the presence of SBE-β-CD and, for this reason, are not analysed in this work.

The ^1^H NMR signals from SBE-β-CD are grouped into four multiplets (A–D) which may be assigned based on [18]. As can be seen in Figure 3b,d, these signals are very broad, which is caused by the random substitution of this CD. In fact, SBE-β-CD is a mixture of CDs substituted with SBE groups to a different extent, and each proton is characterised by a slightly different chemical shift, depending on the exact structure of the molecule in which it is placed. Consequently, the signal superposition from a variety of differently substituted CDs results in a severe peak broadening. In the literature, there are many examples of the application of Rotating-frame nuclear Overhauser Effect SpectroscopY (ROESY) to elucidate the structure of complexes with native unsubstituted CDs (α-, β-, γ-CD) [19]. However, due to the random substitution pattern of SBE-β-CD, both the H-3 and H-5 protons of a CD contribute, together with other protons, to a severely broadened multiplet B, significantly reducing the sensitivity of the experiment. Additionally, the ROESY spectrum contains numerous strong artifacts (Figure S3a), leading to contamination by signals that cannot be interpreted as cross-peaks. Nevertheless, despite the lack of reliable results from ROESY, the proposal of the most probable structure of the formed complex (Figure 2) became possible thanks to the combination of computational techniques: molecular docking and molecular dynamics [13].

The most promising sample containing a solid acid, i.e., ceftobiprole/maleic acid/SBE-β-CD in a molar ratio of 1:25:4, was investigated by NMR (Figure 3d), together with its CD-free counterpart with the same maleic acid ratio of 1:25 (Figure 3c). The presence of maleic acid resulted in the observation of one additional singlet in the NMR spectrum, accompanied by two intense sideband doublets (^13^C satellites). The ratio of the signal integrals confirms the preservation of the initial molar ratio of ceftobiprole to maleic acid of 1:25, which proves that maleic acid does not sublimate during the freeze-drying process and remains in the sample.

As can be seen in Figure 3c,d, the chemical shift of the ceftobiprole H1′ proton changed from 7.07 ppm (ceftobiprole/maleic acid 1:25) to 7.22 ppm (1:25:4 with SBE-β-CD), which may indicate the formation of a complex. An additional interesting observation is that the value 7.07 ppm, characterising the system with maleic acid, is much higher than the previously discussed 6.91 ppm, determined for ceftobiprole freeze-dried in 0.1 M HCOOH. This difference, in turn, can be attributed to the pH difference between these two samples. The reversal of acid dissociation may cause distortion of the conjugated double bonds system in which it participates, thus resulting a change in δ for the proton belonging to the same system. For the same reasons as before, the ROESY spectrum was difficult to interpret due to the presence of many artifacts, which were, among others, a consequence of the random SBE-β-CD substitution, and the high variability of the signal dynamics in the spectrum (Figure S3b).

Another approach to investigate the complex formation using NMR is the use of Diffusion-Ordered SpectroscopY (DOSY) [19]. Based on chemical shifts, each individual signal can be assigned to a proper species present in the analysed sample solution, thus allowing us to determine its diffusion coefficient D. The value of D depends on the hydrodynamic radius r of the analysed species, temperature T, and on the dynamic viscosity η of the solution:

(1) $$D=\frac{k_{B}T}{6\pi \;\eta \;r}$$

Here, $k_{B}$ stands for the Boltzmann constant. The solutions used for the DOSY measurements should be appropriately diluted to avoid the viscosity-related issues. Moreover, to properly compare the DOSY spectra between the samples with and without SBE-β-CD, it must be checked whether the viscosities of these two samples are similar. This, in turn, may be estimated from the comparison of D for the species not taking place in complex formation, preferably water (HDO) and maleic acid with caution, which can possibly form a complex with CD to some extent. It turned out that the condition of similar viscosity was met when the 1:25:4 system at a ceftobiprole concentration of 1.9 mM was compared with its non-CD counterpart at a concentration corresponding to 7.5 mM of ceftobiprole. In the spectra acquired for these two solutions, HDO was characterised by D of 18.47 × 10^−10^ m^2^/s and 18.42 × 10^−10^ m^2^/s, respectively, while maleic acid exhibited D of 7.19 × 10^−10^ m^2^/s and 7.11 × 10^−10^ m^2^/s, respectively.

According to the DOSY spectrum of the system without SBE-β-CD (ceftobiprole/maleic acid 1:25), uncomplexed ceftobiprole is characterised by $D_{CB}\approx \;$3.16 × 10^−10^ m^2^/s (Figure S2a). Meanwhile, the DOSY of the sample with SBE-β-CD provides two values of the diffusion coefficient: $D_{CB-CD}\approx$ 2.68 × 10^−10^ m^2^/s for the complexed form of ceftobiprole and $D_{CD}\approx$ 1.79 × 10^−10^ m^2^/s for SBE-β-CD (Figure S2b). It is a generally accepted practice to approximate that D is the same for the CD in a complex and for free CD. Assuming a quick exchange regime, to calculate the molar fraction $MF_{CB}$ of ceftobiprole molecules being complexed in relation to all ceftobiprole molecules in the studied solution, one can take advantage of the equation stating that $D_{CB-CD}$ is the weighted average of $D_{CB}$ and $D_{CD}$:

(2) $$D_{CB-CD}=MF_{CB}D_{CB}+(1-MF_{CB})D_{CD}$$

The above equation may be interpreted as follows: when ceftobiprole is uncomplexed, it diffuses faster, according to $D_{CB}\approx$ 3.16 × 10^−10^ m^2^/s, but when it is placed inside the CD cavity, the diffusion of this large supramolecular system is lower, $D_{CD}\approx$ 1.79 × 10^−10^ m^2^/s. The complexation equilibrium is dynamic, and can be interpreted as a statistical ceftobiprole molecule spending $MF_{CB}$ proportion of time in a complex, and $\left(1-MF_{CB}\right)$ fraction of time alone, resulting in overall $D_{CB-CD}$ being the weighted average. Solving Equation (2) yields $MF_{CB}$ ≈ 0.35. Based on this finding, the equilibrium constant K may be determined as follows:

(3) $$K=\frac{\left[CB:CD\right]}{\left[CD\right][CB]}=\frac{MF_{CB}}{\left(c_{CD}-MF_{CB}c_{CB})(1-MF_{CB}\right)}$$

Here, the unknown equilibrium concentrations ([CB:CD]—of the complex, $\left[CD\right]$—of the free CD, and [CB]—of the uncomplexed ceftobiprole) have been expressed in terms of the known values: $MF_{CB}$, determined based on the DOSY experiment, as well as $c_{CB}$ = 1.917 mM and $c_{CD}$ = 7.667 mM—analytical concentrations of the ingredients in the studied solution. The obtained value, 77 M^−1^, should be considered as semi-quantitative, due to the large uncertainty of reading the values for D from DOSY spectra, and owing to the application of simplifying assumptions.

Nevertheless, the knowledge of K allows us to estimate how the molar fraction would change for different dilutions of the sample or for different molar ratios of ceftobiprole to SBE-β-CD in the initial solution to be freeze-dried. It is a very beneficial possibility, since due to the viscosity-related issues, the solutions for DOSY experiments had been appropriately diluted to enable obtaining the reliable results. Now, let us consider solutions of concentrations near to the solubility limit (12 mg/mL of ceftobiprole, i.e., 22.5 mM). For the 1:25:4 complex, when $c_{CB}$ = 22.5 mM and $c_{CD}$ = 90 mM (according to the molar ratio 1:4), solving Equation (3) with K = 77 M^−1^ yields $MF_{CB}$ = 0.845, which means that when the solution is near saturation, 85% of ceftobiprole molecules are in the complex with SBE-β-CD. When the molar ratio is 1:25:2, then $c_{CD}$ = 45 mM, resulting in $MF_{CB}$ = 0.693. This means that increasing the amount of SBE-β-CD in the sample results in higher efficiency of complex formation and proves that setting the molar ratio between ceftobipriole and SBE-β-CD to 1:4 was a good choice.

### 2.4. Characterisation in the Solid State

**Figure 4:**
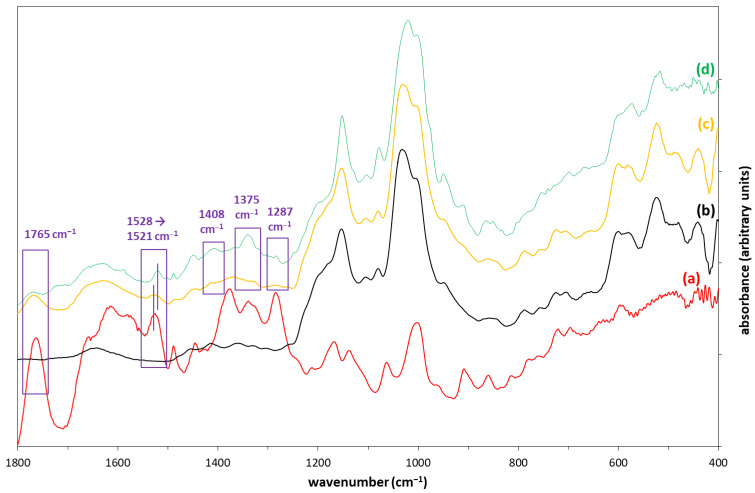
ATR-FTIR spectra in the range 1800–400 cm^−1^ recorded for the following powders: (**a**) ceftobiprole freeze-dried in 0.1 M HCOOH; (**b**) SBE-β-CD freeze-dried in 0.1 M HCOOH; (**c**) 1:2 physical mixture of (**a**,**b**); (**d**) 1:2 complex of ceftobiprole with SBE-β-CD, freeze-dried together in 0.1 M HCOOH.

The prepared solid samples were studied using ATR-FTIR and XRPD, which are the most common solid-state techniques for studying CD complex formation [20] and controlling their physical stability [21]. The measurements were limited to the samples freeze-dried with HCOOH and maleic acid, as they were the most promising ones. The comparison was made between the ceftobiprole/SBE-β-CD sample freeze-dried in 0.1 M HCOOH and the physical mixture of both components freeze-dried separately in 0.1 M HCOOH. In the range of 1800–400 cm^−1^, the following differences can be observed between the complex and the physical mixture (Figure 4d vs. Figure 4c): a significant decrease in the peak intensity at 1765 cm^−1^ (vibrations of the carbonyl group of ceftobiprole), a shift of the peak maximum from 1528 cm^−1^ (for ceftobiprole and physical mixture) to 1521 cm^−1^ (for a complex), an increase in the intensity at 1408 cm^−1^, 1375 cm^−1^ and 1287 cm^−1^. According to Mura [22], the differences in the peak intensity or the maximum wavenumber may be due to the formation of an inclusion complex between the guest and the CD.

The measurements were also performed for samples of ceftobiprole freeze-dried with maleic acid, with and without the addition of SBE-β-CD. The peaks corresponding to maleic acid, which was present in a 25-fold molar excess, are overlapped on the peaks of ceftobiprole, thus hiding them. These two molecules are characterised by the same function groups: a carboxyl group and a C=C double bond, which causes vibrations at similar frequencies. It follows that the analysis of ternary systems with a significant excess of one component does not allow for drawing constructive conclusions about the formation of a complex between an API and a CD using the ATR-FTIR method.

**Figure 5:**
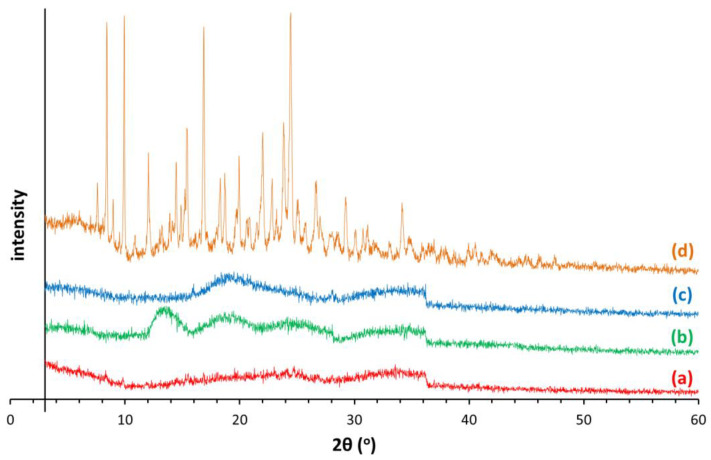
Diffraction patterns recorded for the following powders after specified periods of storage: (**a**) ceftobiprole freeze-dried in 0.1 M HCOOH (3 months); (**b**) 1:2 complex of ceftobiprole with SBE-β-CD freeze-dried in 0.1 M HCOOH (3 months); (**c**) 1:25:4 complex of ceftobiprole, maleic acid and SBE-β-CD freeze-dried in water (8 months); and (**d**) unprocessed ceftobiprole.

The powder diffraction patterns of the freeze-dried samples (both with formic and maleic acid) do not contain any sharp peaks, which shows that the samples are amorphous (Figure 5a–c), while the sample of pure ceftobiprole taken for this study was initially crystalline (Figure 5d). This means that after the freeze-drying process, the individual components do not exist in their crystalline forms. This may be attributed to the overcoming of the intermolecular attraction between the molecules of the same compound as a result of freeze-drying. However, due to the fact that each analysed lyophilised sample is strongly amorphised, and therefore the diffraction patterns of the samples with and without SBE-β-CD are very similar, the XRPD technique could not be used to confirm the formation of the inclusion complex. On the other hand, XRPD allows for monitoring the physical stability of the prepared samples by controlling the tested samples at specific time intervals. The lack of distinct, sharp peaks (Figure 5) proves that the amorphous nature of the samples was preserved and that the undesired recrystallisation process did not occur to a detectable extent.

### 2.5. Development of a Dosage Form for Parenteral Administration

The high molecular weight of ceftobiprole and its molecular structure cause poor solubility in water and limited permeability through biological membranes. Therefore, ceftobiprole is not suitable for oral administration [23] and hence is used in intravenous form as a prodrug—ceftobiprole medocaril, which after administration is converted to the active form of the drug. The low pH of the ceftobiprole/maleic acid/SBE-β-CD 1:25:4 solution (≈1.5) makes it unsuitable for parenteral administration. Due to a pH value of blood plasma of approximately 7.4, solutions for infusion should have a pH in the range of 4.0–7.5 [24]. Thanks to the fact that maleic acid is diprotic and has a second pK_a_ of about 6.1, the choice of this acid was also appropriate in another respect. Namely, it proved helpful in obtaining the correct pH, because buffering the pH of the solution at about 6 makes the neutralisation process of maleic acid less susceptible to small fluctuations in the amount of base added and prevents a shift to a basic pH.

Initially, the pH of the sample was raised to 6 using 10 M NaOH, which caused partial precipitation in the sample. HPLC analysis of the centrifuged solution showed that the solubility of ceftobiprole had decreased to 6.5 mg/mL from the initial 14 mg/mL. Interestingly, although the contact of ceftobiprole with OH^−^ ions was very short (only until these ions were neutralised by H^+^ from maleic acid), it was long enough to cause chemical degradation of ceftobiprole. The content of BDP-1and BDP-2, open-ring ceftobiprole and a lactone, was determined to be 24% in total. Neutralisation with a more dilute (2 M) base still led to an increase in BDPs.

Another approach is to use sodium hydrogencarbonate (NaHCO_3_), which allows the solution to be neutralised without introducing OH^−^ ions, which degrade the sample. Instead, hydrogencarbonate anions combine with protons to form water and carbon dioxide:

(4) $$H^{+}+HCO_{3}^{-}\to H_{2}O+CO_{2}↑$$

During the addition of 1 M NaHCO_3_ to the system, CO_2_ bubbles were released intensively. Neutralisation caused the addition of Na^+^ cations to the system, which added to the Na^+^ ions coming from the sodium salt of SBE-β-CD. The volumes of solutions to obtain a final delivery system of ceftobiprole were selected so that the sample was completely dissolved and centrifugation was not necessary (detailed information is given in Section 3.3). A 20% (*w*/*w*) aqueous solution of the system was prepared, and then 1 M NaHCO_3_ was added to obtain a pH between 4.5 and 5.0. According to the obtained chromatograms, the solubility of ceftobiprole reached 6.0 mg/mL immediately after sample preparation and remained stable over time, decreasing in the solution by 3% within 20 h at 25 °C.

According to the Zevtera Summary of Product Characteristics [17], each vial of this medicinal product contains a single recommended dose of 500 mg ceftobiprole (as 666.6 mg of ceftobiprole medocaril sodium), together with two excipients: citric acid monohydrate and sodium hydroxide, used for pH adjustment. The lyophilised powder should be reconstituted with sterile water for injections or 5% dextrose. The pH of the reconstituted solution is 4.5 to 5.5. Immediate further dilution of the reconstituted solution is recommended. However, if this is not possible, the reconstituted solution can be stored at room temperature for up to 1 h or in a refrigerator for up to 24 h. For adults and children ≥ 12 years of age, the concentration of ceftobiprole in the infusion solution is 2 mg/mL. Considering the ceftobiprole concentration and pH value obtained after the addition of 1 M NaHCO_3_ solution, the comparison of the newly developed drug delivery system with the commercially available drug product demonstrates that both offer similar concentrations of ceftobiprole and pH.

## 3. Materials and Methods

### 3.1. Materials

Ceftobiprole was purchased from BenchChem (Austin, TX, USA). SBE-β-CD (product number CY-2041.2) was bought from CycloLab (Budapest, Hungary). The molar mass of SBE-β-CD used in this study (2294.1 g/mol) was calculated knowing the DS = 6.5 and water content of 5.7%. The deionised water was obtained from a Direct-Q 3 UV Millipore by Merck (Darmstadt, Germany).

The following reagents were used in this study: maleic acid (for synthesis, 99.9%, Merck, Darmstadt, Germany), citric acid monohydrate (puriss. p.a., ≥99.5%, Sigma-Aldrich, St. Louis, MO, USA), *p*-toluenesulfonic acid monohydrate (ReagentPlus, ≥98%, Sigma-Aldrich, St. Louis, MO, USA), formic acid (≥98%, Merck, Darmstadt, Germany), hydrochloric acid (pure p.a., 35–38%, POCH, Gliwice, Poland), ammonium acetate (reag. Ph. Eur. for HPLC/UV studies or LiChropour for mass spectrometry, both from Merck, Darmstadt, Germany), acetic acid (pharma grade, 99.9%, AppliChem, Darmstadt, Germany, for HPLC/UV studies, or 100% LiChropour for LC-MS, Merck, Darmstadt, Germany), and acetonitrile (gradient grade from Honeywell, Seelze, Germany, for HPLC/UV studies, or hypergrade for LC-MS from Merck, Darmstadt, Germany).

### 3.2. Freeze-Drying Experiments

Freeze-drying experiments were performed using two devices: Alpha 1-2 LDplus (Martin Christ, Osterode am Harz, Germany) or FreeZone 2.5 L (Labconco, Kansas City, MO, USA). A total of 0.1 mmol (53.5 mg) of ceftobiprole was dissolved in 600 mL of a solvent while other substances were weighed according to an appropriate molar ratio. The resulting concentration of ceftobiprole in the solution to be freeze-dried (0.09 mg/mL) is below its solubility limit in 0.1 M HCOOH (0.14 mg/mL [13]). In some experiments, the weights and solvent volume were proportionally decreased while maintaining the desired molar concentrations of ingredients. The freshly prepared solutions were sonicated for 15 min and magnetically stirred for 3 h. Then, an equilibrated solution was poured into round-bottom flasks, which were then attached to the valves of freeze-drying equipment. The process was continued until the last piece of ice disappeared (generally between 24 h and 40 h).

### 3.3. Preparation of Ceftobiprole/Maleic Acid/SBE-β-CD (1:25:4) System at pH ~ 4.5

In order to obtain a drug form with a pH of about 4.5 for the ceftobiprole/maleic acid/SBE-β-CD 1:25:4 system, the following procedure was established: 23.6 mg of the system (containing 1 mg of ceftobiprole) was weighed, 100 μL of water was added to achieve complete dissolution, and then 50 μL of 1 M NaHCO_3_ was added to obtain the appropriate pH.

### 3.4. Chromatographic Analysis

The HPLC/UV analysis was performed according to the method developed in [14], using a Shimadzu Nexera-i LC-2040C (Kyoto, Japan) liquid chromatograph with a UV–VIS detector, equipped with LabSolutions 5.87 data processing software. The LC-MS/MS experiments were performed using the same equipment and methodology as described in [14], with one modification: to acquire a full mass spectrum of SBE-β-CD, the range of detected *m*/*z* was broadened to 2500.

### 3.5. NMR Studies

The NMR experiments were performed on a Varian vnmrs 600 MHz spectrometer (Agilent Technologies, Santa Clara, CA, USA) using Auto XID (inverse configuration) probe head with resonant frequencies 599.8 MHz for ^1^H and 150.8 MHz for ^13^C measurements. All spectra were run in D_2_O solutions at room temperature (25 °C). The ^1^H NMR spectra and the ^1^H dimension in two-dimensional (2D) heteronuclear spectra were referenced to the solvent (D_2_O, δ_H_ = 4.64 ppm). For both the acquisition and processing of data, the standard parameters and procedures were applied. The duration of the 90° pulse for ^1^H was between 6.75 and 6.90 μs. The spectral width for proton spectra was set to 5000 Hz. The time of relaxation delay in the experiments was set to 1 s, if not stated otherwise.

The 1D ^1^H NMR spectra were obtained using a pulse duration of 2.5 μs, which corresponds to a tilt angle of approximately 32.6°. In each single experiment, 32 scans were recorded, each with 16,384 complex data points. The ^1^H–^1^H couplings through space were determined using the ROESY pulse sequence with 200 increments applied for t_1_, each comprising four scans with 1024 complex points and 300 ms spinlock mixing time. The formation of a complex between ceftobiprole and SBE-β-CD was evidenced by the changes in diffusion coefficients determined in the pseudo-2D DOSY experiments using ONESHOT sequence [25]. In these studies, 16 increments consisting of 64 scans were performed. A relaxation time of 2 s, a diffusion time of 200 ms, a total diffusion encoding gradient duration of 2 ms, and 16 values of the diffusion encoding gradient incremented from 3 to 25 G/cm were applied. Processing was carried out using the OpenVnmrJ 3.1A software with the option of correction for spatially non-uniform pulsed field gradients [26].

### 3.6. ATR-FTIR Studies

A Nicolet iS5 FT-IR spectrometer (Thermo Scientific, Waltham, MA, USA) with an attenuated total reflectance (ATR) attachment with diamond crystal was used to collect spectra in the region from 4000 to 400 cm^−1^. Spectra were recorded using 32 scans with a resolution of 4 cm^−1^. Data were collected using OMNIC 9.8.

### 3.7. XRPD Studies

XRPD studies were performed using a D8 Advance diffractometer (Bruker, Billerica, MA, USA) with a copper-type tube as the X-ray source and a scintillation detector. Samples were analysed within the standard angular range of 3° < 2θ < 60°. Diffraction measurements were carried out using the Bragg–Brentano method with a step size of 0.021° with a time per step of 2.5 s.

## 4. Conclusions

In this study, novel CD-based delivery systems for ceftobiprole were developed. SBE-β-CD was selected as the key ingredient based on the results of molecular dynamics simulation. Experiments were conducted using acids of varying strength and volatility. When 0.1 M formic acid was used to lower the pH of the solution prior to freeze-drying, it left the solution almost completely during sublimation. The use of 0.1 M formic acid and SBE-β-CD significantly increased the solubility of ceftobiprole from about 0.1 mg/mL to 3.4 mg/mL. A possible mechanism was proposed to explain this enhancement in solubility. Lowering the pH inhibits the formation of extensive molecular aggregates and instead promotes the isolation of individual molecules. The weakening of the interactions between ceftobiprole molecules facilitated its interaction with SBE-β-CD and the formation of inclusion complexes, which further increased its solubility. Complex formation was confirmed by ^1^H NMR and ATR-IR spectroscopy.

When solid acids were used, they did not sublimate during lyophilisation but remained in the sample, thus forming a ternary system. After dissolving the lyophilisate, the solution had a strongly acidic pH, which led to a further improvement in the solubility of ceftobiprole. However, the use of citric acid resulted in high hygroscopicity, while tosylic acid caused an unacceptable rate of chemical degradation in the solid state. The most favourable results combining high solubility, low levels of DPs and good long-term stability were obtained with a ceftobiprole/maleic acid/SBE-β-CD system in a molar ratio of 1:25:4. This formulation increased the solubility of ceftobiprole nearly 300-fold, from 0.05 mg/mL to approximately 14 mg/mL. Moreover, the use of NaHCO_3_ allowed us to increase the pH of a sample up to a value acceptable for parenteral administration, without inducing alkaline degradation. The developed system offers similar physicochemical capabilities to the commercially available drug Zevtera, which contains ceftobiprole in the form of a medocaril prodrug.

The presented methodology can be extended to other basic or amphoteric compounds that are thermally labile but chemically stable in acidic solutions. The set of recommended studies includes (i) theoretical calculations: the selection of the optimal CD based on the most negative free Gibbs energy for inclusion of the model compound; (ii) ionisation of the compound: the application of appropriate volatile and non-volatile acids to ionise the model compound; (iii) complex optimisation: the preparation of the inclusion complex, including freeze-drying of samples; (iv) solubility studies: checking the solubility of the obtained complexes over time; (v) IR spectroscopy and X-ray diffractometry: confirming the formation of the complex or amorphisation of the sample; and (vi) stability assessment: analysis of the physical and chemical stability of the complexes.

## Figures and Tables

**Table 1:** Summary of the results of studies on new drug delivery systems for ceftobiprole in terms of solubility and chemical stability.

System		pH		Solubility of Ceftobiprole for Freeze-Dried Samples in a Preliminary Study		Stability in Solid State	DPs Detected ^1^ (Estimated Sum in a Preliminary Study)
		Before Freeze-Drying	After Freeze-Drying	Without SBE-β-CD (mg/mL)	With SBE-β-CD (mg/mL)	Solubility (mg/mL) After *n* Months	
volatile acids	ceftobiprole/SBE-β-CD 1:2 in 0.1 M **HCOOH**	2.3	4.0	2.1	3.4	*not selected* *for further study* ^2^	NDP-1, ODP-1, BDP-1, ODP-2, PDP-1 (1.2%)
	ceftobiprole/SBE-β-CD 1:2 in 0.01 M **HCl**	2.0	1.0	*not tested*	1.0	*not selected* *for further study* ^2^	numerous, see Figure 1g
non-volatile acids	ceftobiprole/**citric acid**/SBE-β-CD in H_2_O 1:100:1	2.4	1.5	*not tested*	8.3	7.3 (6 m), 5.1 (9 m)	NDP-1, ODP-1, ADP-1, BDP-1, ADP-2, ODP-2, ADP-0 (6.8%)
	ceftobiprole/**tosylic acid**/SBE-β-CD in H_2_O 1:25:2	2.4	0.5	*not tested*	21.3	13.8 (1.5 m), 11.3 (6 m), 6.7 (7 m), 4.5 (8.5 m)	NDP-1, ODP-1, ADP-1, BDP-1, ADP-2, ODP-2, ADP-0, ADP-4, ADP-5, PDP-1, PDP-2, ADP-6 and unknown DPs (8.6%)
	ceftobiprole/**maleic acid**/SBE-β-CD in H_2_O 1:5:4	3.1	3.0	*not tested*	0.7	*not selected* *for further study* ^2^	NDP-1, ODP-1, BDP-1, ODP-2, PDP-1, PDP-2 (1.0%)
	ceftobiprole/**maleic acid**/SBE-β-CD in H_2_O 1:15:4	2.7	2.0	1.1	1.4	*not selected* *for further study* ^2^	NDP-1, ODP-1, BDP-1, ODP-2, ADP-0, PDP-1, PDP-2 (1.4%)
	ceftobiprole/**maleic acid**/SBE-β-CD in H_2_O 1:20:4	2.6	1.5	1.3	10.6	*not selected* *for further study* ^2^	NDP-1, ODP-1, BDP-1, ODP-2, ADP-0, PDP-1, PDP-2 (1.8%)
	ceftobiprole/**maleic acid**/SBE-β-CD in H_2_O 1:25:4	2.5	1.5	3.7	14.0	12.4 (6 m), 11.3 (8 m)	NDP-1, ODP-1, BDP-1, ODP-2, ADP-0, PDP-1, PDP-2 (2.4%)

^1^ The abbreviations for degradation products (DPs) come from the conditions in which they predominantly form: acidic (ADPs), basic (BDPs), oxidative (ODPs) and photolytic (PDPs). The proposed structural formulas for individual identified DPs were presented in [14]. The sum of DP content is estimated based on the assumption that the response factors of DPs and ceftobiprole are equal. ^2^ The reason for discontinuing the study was the unsatisfactory improvement in the solubility of ceftobiprole or too rapid degradation.

## Data Availability

Data is contained within the article and Supplementary Material.

## Supplementary Materials

The supporting information can be downloaded at: https://www.mdpi.com/article/10.3390/ijms26135953/s1.
