# Supplementary material for: Development of a Cyclodextrin-Based Drug Delivery System to Improve the Physicochemical Properties of Ceftobiprole as a Model Antibiotic
Source: Int J Mol Sci. 2025 Jun 20;26(13):5953. doi: 10.3390/ijms26135953 (PMC12249915; doi:10.3390/ijms26135953)
Supplement: Supplementary file 1 [file ijms-26-05953-s001.zip › ijms-3675701-supplementary.pdf]

Supplementary Material for:

Development of a Cyclodextrin-Based Drug Delivery System to Improve the Physicochemical Properties of Ceftobiprole as a Model Antibiotic

Dariusz Boczar [1,*], Wojciech Bocian [2], Jerzy Sitkowski [3], Karolina Pioruńska [3] and Katarzyna Michalska [1,*]

[1] Department of Synthetic Drugs, National Medicines Institute, Chełmska 30/34, 00-725 Warsaw, Poland
[2] Laboratory for Analysis of Bioactive Compounds, Institute of Organic Chemistry, Polish Academy of Sciences, Kasprzaka 44/52, 01-224 Warsaw, Poland; wo.bocian@gmail.com
[3] Falsified Medicines and Medical Devices Department, National Medicines Institute, Chełmska 30/34, 00-725 Warsaw, Poland; j.sitkowski@nil.gov.pl (J.S.); k.piorunska@nil.gov.pl (K.P.)
* Correspondence: d.boczar@nil.gov.pl (D.B.); k.michalska@nil.gov.pl (K.M.)

2.2. Solubility and chemical stability studies using HPLC

LC-MS characterisation of SBE-β-CD

When the prepared cyclodextrin-based delivery systems were analysed using the optimised chromatographic method to determine ceftobiprole and its related substances, no additional peaks resulting from SBE-β-CD were detected by UV detector at 320 nm due to the lack of an appropriate chromophore in the molecule of this CD. On the other hand, the LC-MS analysis performed with the same method showed that SBE-β-CD eluted from the column over a range of retention times from 2 to 11 min. Figure S1-a presents the average of mass spectra recorded in that period. Peaks are detected mainly for m/z up to 1500 Da and, according to their isotopic profiles, refer to doubly or even triply charged species. However, performing the deconvolution procedure within the Data Analysis software allowed to simulate how the spectrum would look like if the all the species were singly charged (Figure S1-b). As can be seen in the deconvoluted spectrum, each group of peaks refers to a specific fraction of SBE-β-CD of the same DS, and individual peaks within each group differ by the number of $NH4^+$ cations in the detected cluster, further denoted as *x*. Each SBE group introduces the charge of −1, and therefore the commercially available SBE-β-CD is marketed as a sodium salt, since Na^+ cations are required to neutralise the negative charge of SBE groups. However, when SBE-β-CD elutes from the chromatographic column together with the mobile phase containing ammonium acetate, the anions of SBE-β-CD may combine with NH_4^+ cations from the mobile phase in the ion source, thus forming adducts of different stoichiometries. For this reason, each cluster composed of a proper species from SBE-β-CD characterised with a specified DS and accompanied with *x* $NH4^+$ cations may be described by the general molecular formula: $C_{42}H_{71}O_{35}(C_4H_8O_3)DS(NH_3)x$. This formula allows to calculate the monoisotopic mass expressed in Da, using the following equation: 1135.377040 + 136.019415 DS + 17.026549 x. The interpretation of mass spectrum presented in Figure S1-b is evidenced in detail in Table S1.

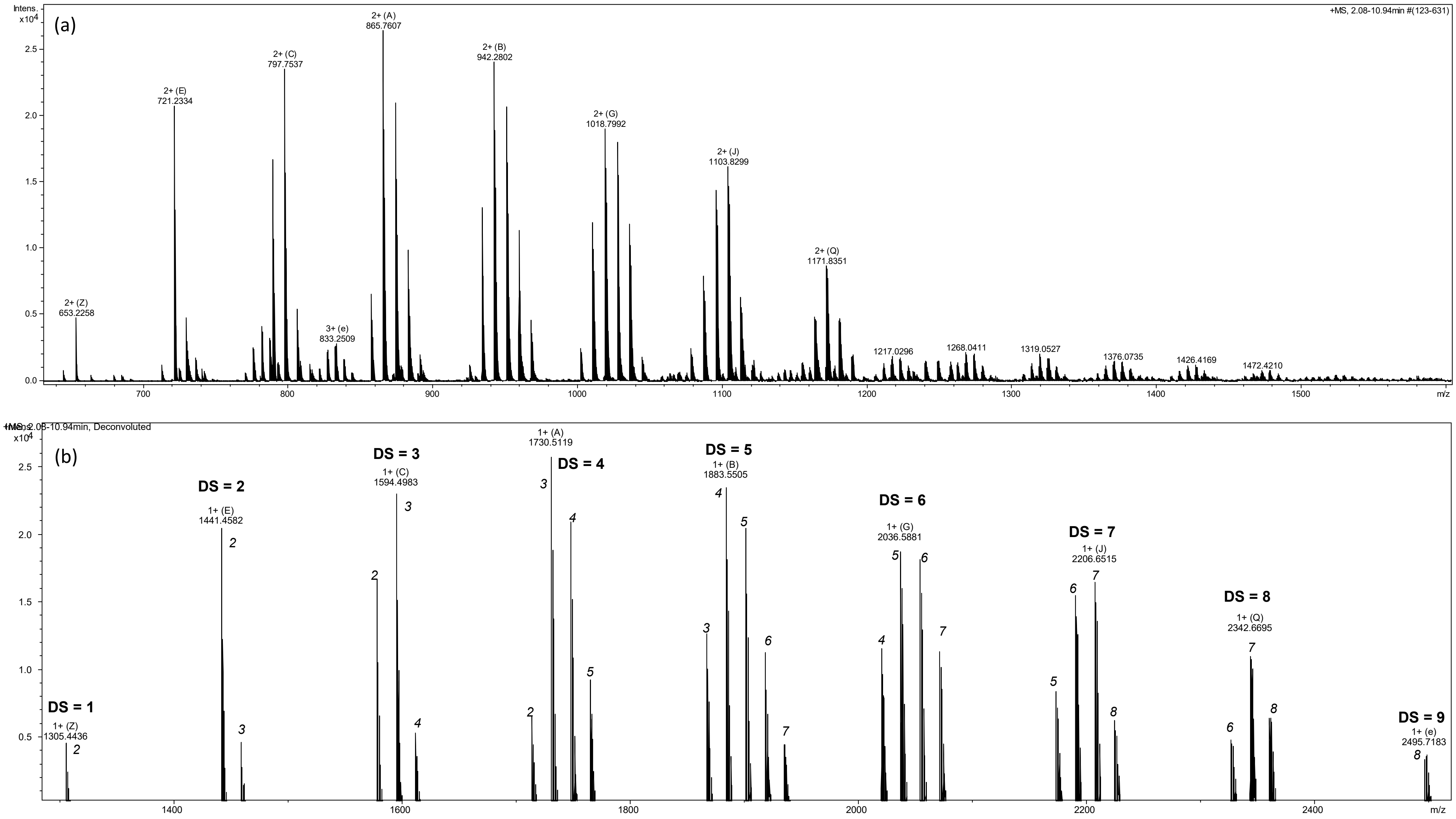

Figure S1. (a) Raw mass spectrum recorded for SBE-β-CD, (b) the deconvoluted mass spectrum of SBE-β-CD. The numbers in italics accompanying each peak refer to the number of NH4+ cations in the detected cluster (x).

Table S1. Interpretation of mass spectrum of SBE-β-CD recorded in LC-MS study.

DS	*x*	Ionic formula	m_{calc}	m_{exp}
1	2	$C_{46}H_{85}O_{38}N_2S$	1305.4496	1305.4436
2	2	$C_{50}H_{93}O_{41}N_2S_2$	1441.4690	1441.4582
2	3	$C_{50}H_{96}O_{41}N_3S_2$	1458.4955	1458.4774
3	2	$C_{54}H_{101}O_{44}N_2S_3$	1577.4884	1577.4724
3	3	$C_{54}H_{104}O_{44}N_3S_3$	1594.5149	1594.4983
3	4	$C_{54}H_{107}O_{44}N_4S_3$	1611.5415	1611.5242
4	2	$C_{58}H_{109}O_{47}N_2S_4$	1713.5078	1713.4868
4	3	$C_{58}H_{112}O_{47}N_3S_4$	1730.5343	1730.5119
4	4	$C_{58}H_{115}O_{47}N_4S_4$	1747.5609	1747.5375
4	5	$C_{58}H_{118}O_{47}N_5S_4$	1764.5874	1764.5628
5	3	$C_{62}H_{120}O_{50}N_3S_5$	1866.5538	1866.5251
5	4	$C_{62}H_{123}O_{50}N_4S_5$	1883.5803	1883.5505
5	5	$C_{62}H_{126}O_{50}N_5S_5$	1900.6069	1900.5759
5	6	$C_{62}H_{129}O_{50}N_6S_5$	1917.6334	1917.6011
5	7	$C_{62}H_{132}O_{50}N_7S_5$	1934.6600	1934.6265
6	4	$C_{66}H_{131}O_{53}N_4S_6$	2019.5997	2019.5623
6	5	$C_{66}H_{134}O_{53}N_5S_6$	2036.6263	2036.5881
6	6	$C_{66}H_{137}O_{53}N_6S_6$	2053.6528	2053.6134
6	7	$C_{66}H_{140}O_{53}N_7S_6$	2070.6794	2070.6381
7	5	$C_{70}H_{142}O_{56}N_5S_7$	2172.6457	2172.6015
7	6	$C_{70}H_{145}O_{56}N_6S_7$	2189.6722	2189.6274
7	7	$C_{70}H_{148}O_{56}N_7S_7$	2206.6988	2206.6515
7	8	$C_{70}H_{151}O_{56}N_8S_7$	2223.7253	2223.6730
8	6	$C_{74}H_{153}O_{59}N_6S_8$	2325.6917	2325.6344
8	7	$C_{74}H_{156}O_{59}N_7S_8$	2342.7182	2342.6695
8	8	$C_{74}H_{159}O_{59}N_8S_8$	2359.7448	2359.6972
9	8	$C_{78}H_{167}O_{62}N_8S_9$	2495.7642	2495.7183

2.3. NMR studies

<u>2D NMR spectra of the developed systems</u>

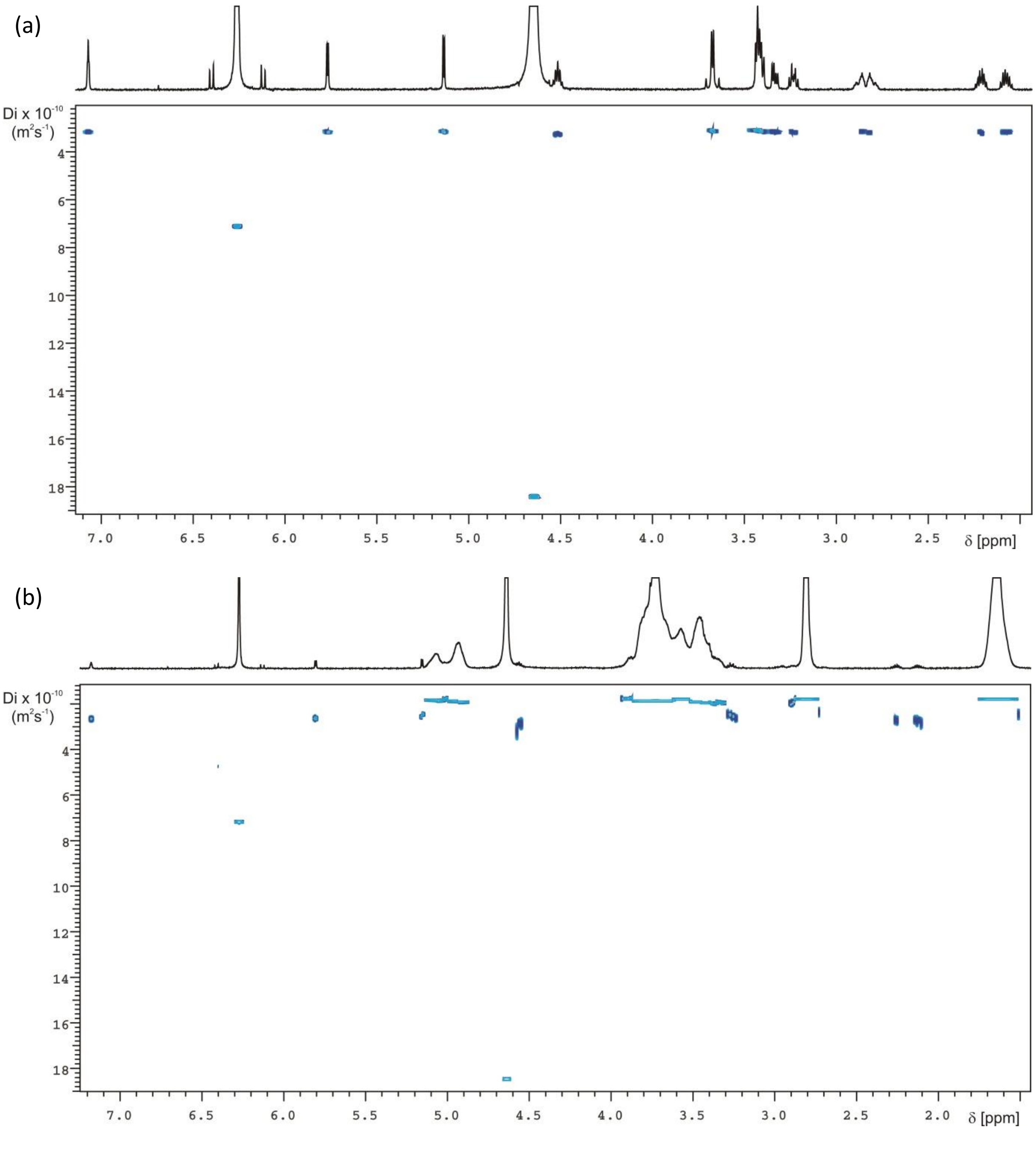

Figure S2. The DOSY spectra recorded in D_2O for the systems: (a) ceftobiprole/maleic acid 1:25 (c_{CB} = 7.5 mM); (b) ceftobiprole/maleic acid/SBE-β-CD 1:25:4 (c_{CB} = 1.9 mM).

(a)

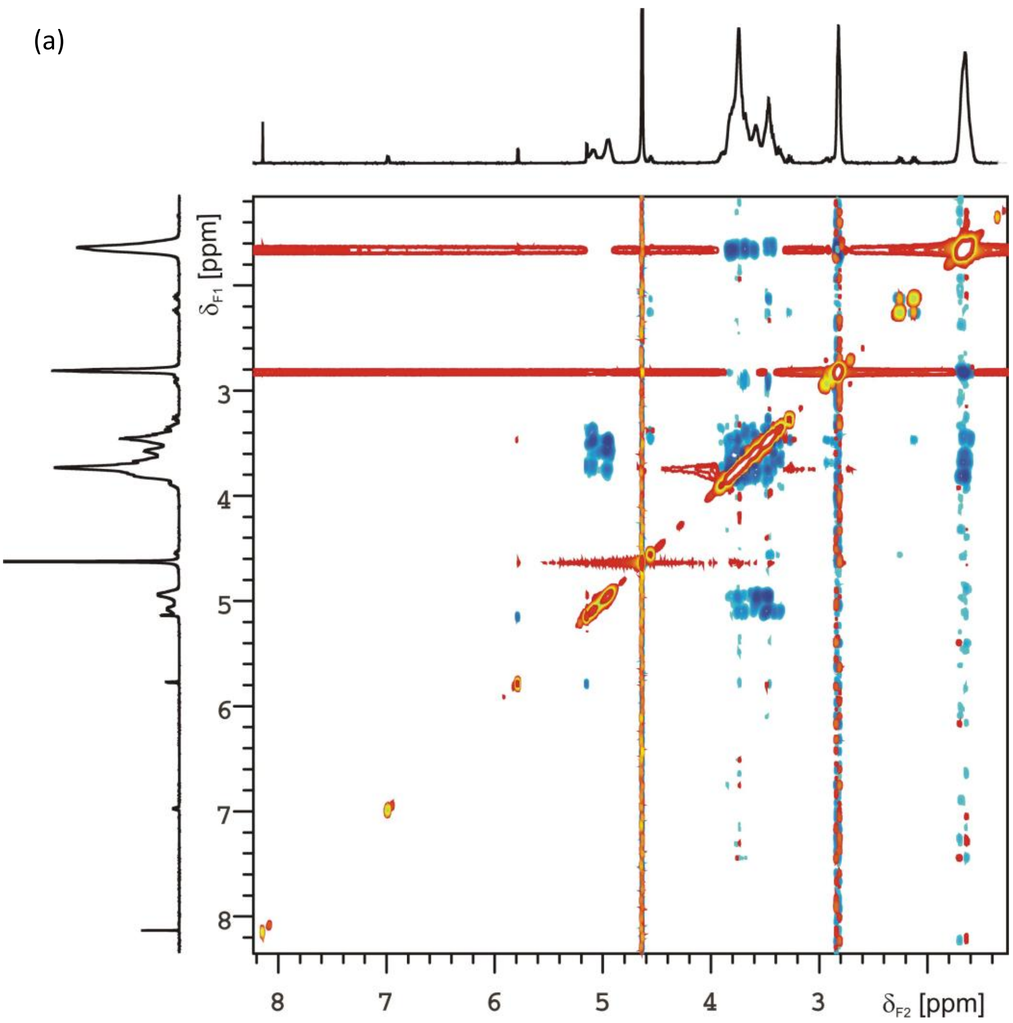

(b)

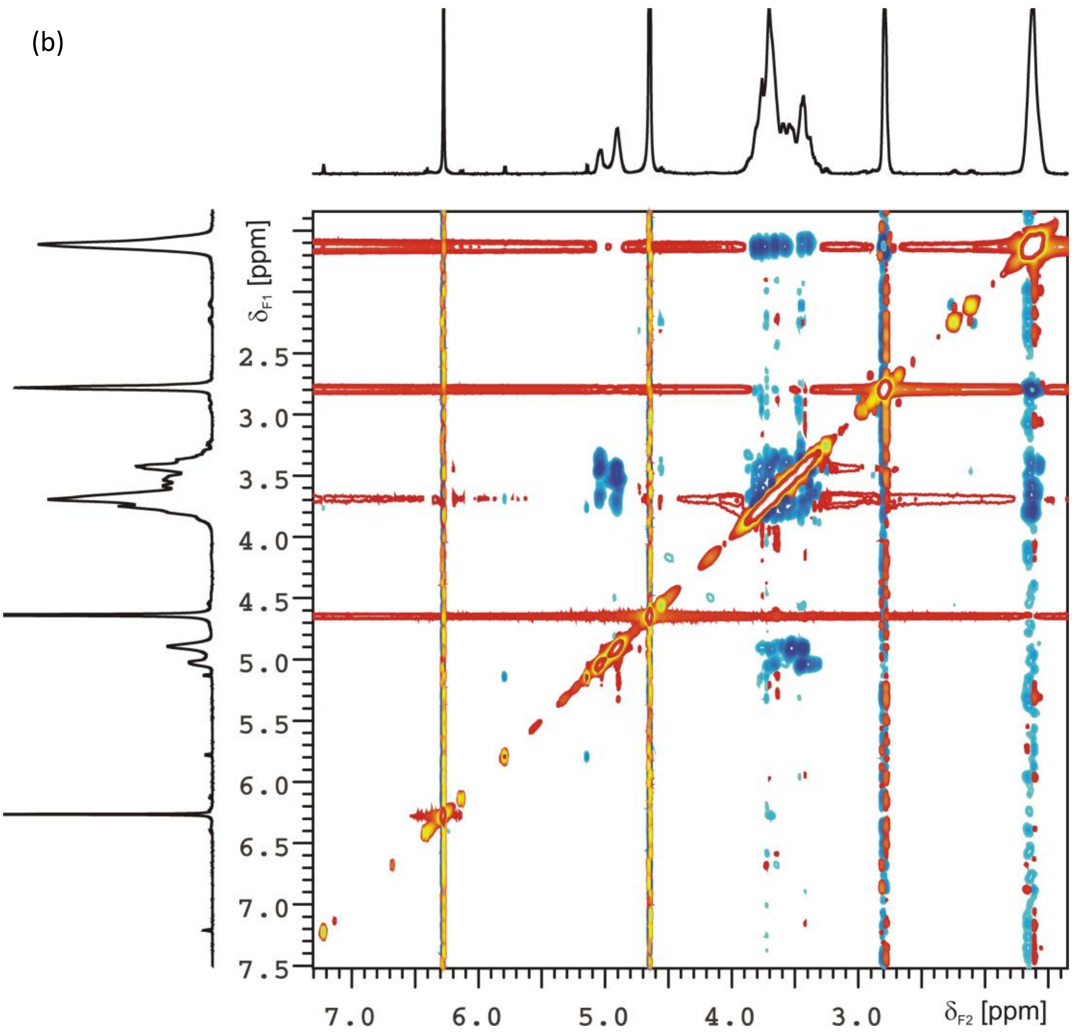

Figure S3. The ROESY spectra in D_2O recorded for the systems: (a) ceftobiprole/SBE-β-CD 1:2 in 0.1 M HCOOH; (b) ceftobiprole/maleic acid/SBE-β-CD 1:25:4. The concentration of ceftobiprole was 4 mg/mL (7.5 mM) in both experiments.
